# COP29: Progresses and challenges to global efforts on the climate crisis

**DOI:** 10.1016/j.xinn.2024.100748

**Published:** 2024-12-03

**Authors:** Jing Wei, Tong Jiang, Philippine Ménager, Dong-Gill Kim, Wenjie Dong

**Affiliations:** 1 School of Atmospheric Sciences, Sun Yat-sen University, Zhuhai 519082, China; 2 Research Institute of Climatic and Environmental Governance/Institute for Disaster Risk Management, School of Geographical Science, Nanjing University of Information Science & Technology, Nanjing 210044, China; 3 Fundación Ecología y Desarrollo, Casco Antiguo, 50001 Zaragoza, Spain; 4 Wondo Genet College of Forestry and Natural Resources, Hawassa University, PO Box 128, Shashemene, Ethiopia

## Main text

**Figure 1:**
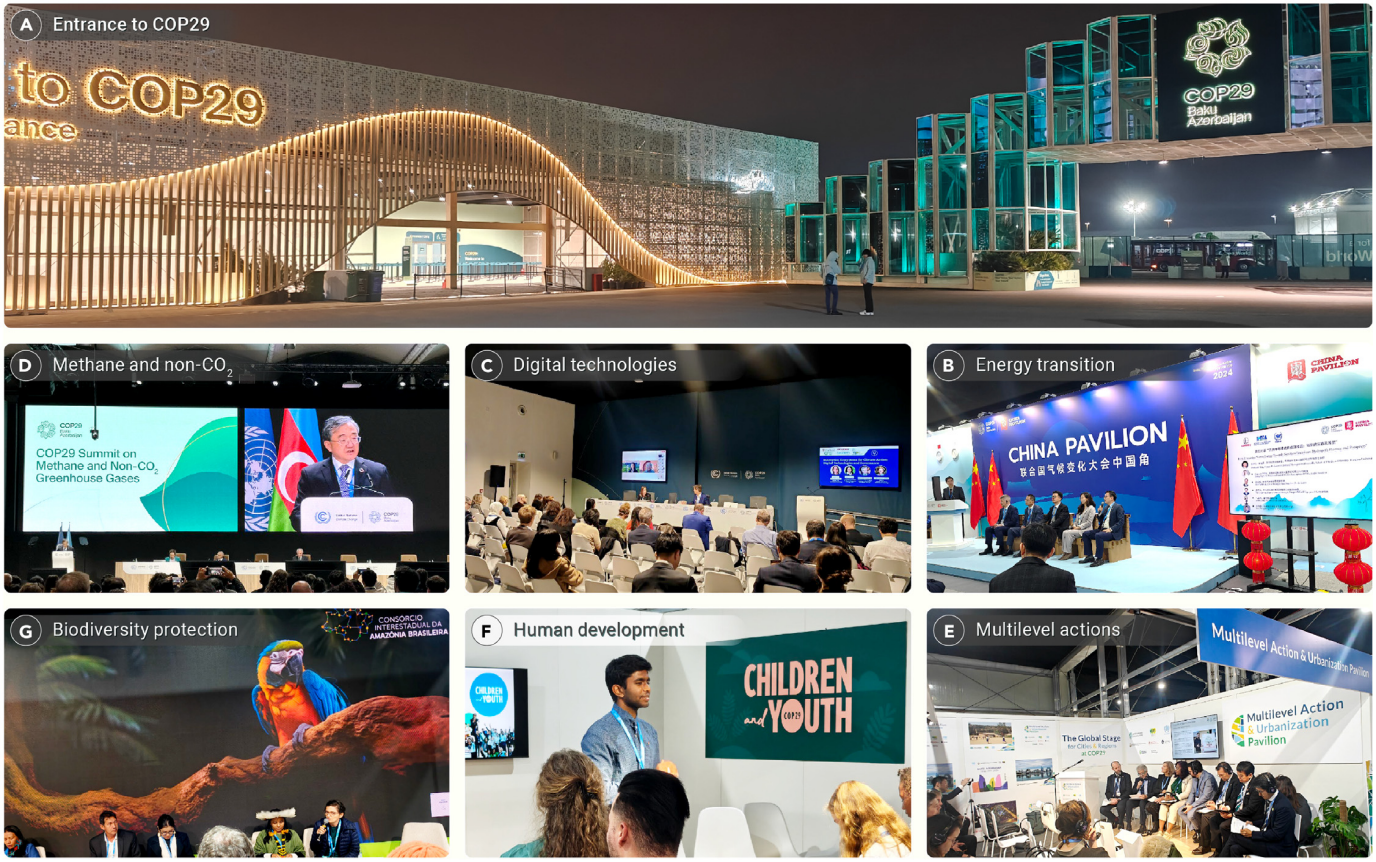
COP29 conference venue and events (A) Entrance to COP29. (B) Chinese specialists talking about the potential application of hydrogen. (C) A side event for advancing digital technologies in climate mitigation and adaption. (D) Summit on mitigation of methane and non-CO_2_ GHGs. (E) Discussions about multilevel actions for climate-positive urbanization. (F) Climate education for children and youth. (G) Legal Amazon Consortium calling for actions to protect the Amazon ecosystem. Photos by Dr. Jing Wei.

On July 22, 2024, the global surface temperature reached a record-breaking 17.16°C, heightening concerns about the feasibility of the 1.5°C limit set by the Paris Agreement. Due to climate change, the intensity of extreme weather events has been surging, resulting in significant losses, damages, and reducing living space for humanity. The 29th Conference of the Parties to the United Nations Framework Convention on Climate Change (UNFCCC COP29; Figure 1A) in Baku, Azerbaijan, from November 11^th^ to 22^nd^, 2024, presented a pivotal opportunity for global nations to unite, enhance climate ambitions, and accelerate climate action.

## Pathway to COP29

The Glasgow Agreement (COP26) in 2021 reaffirmed the 1.5°C limit goal but fell short on concrete financial commitments. COP27 in Egypt established a financial fund for loss and damage but lacked specific implementation details. COP28 in Dubai marked a significant milestone with the finalization of the first global stocktake (GST), which included an explicit reference to phasing out fossil fuels for the first time,^1^ setting the stage for COP29 to address remaining financial issues and implementation measures.

## Progresses of COP29

### NDCs

Under the Paris Agreement, parties are obligated to submit revised nationally determined contributions (NDCs) to the UNFCCC secretariat every 5 years. The G20, responsible for approximately 75% of global greenhouse gas (GHG) emissions, faces significant pressure to enhance its commitments and achieve net-zero targets by 2050. As the next NDC deadline approaches in February 2025, some parties have already announced their intentions at COP29: Denmark aims for a fossil-free energy system by 2050, integrating over 70% of renewable energy into its smart grid, and the United States has set a target of reducing carbon emissions by 50%–52% below the 2005 level by 2030 and quadrupling its international climate finance to $11 billion per year.

### Climate finance

The annual $100 billion climate finance goal committed by developed countries was finally met in 2022. COP29, dubbed the "Finance COP," prioritized the establishment of a new collective quantified goal on climate finance (NCQG). Following this requirement, the Baku Climate Unity Pact was achieved at COP29, calling to provide a minimum of $300 billion annual by 2035 to developing countries for climate mitigation, adaption, loss, and damage, as well as the achievement of next NDCs.

### Global carbon market

The global carbon market plays a crucial role in reducing carbon emissions cost effectively and fostering international cooperation. While Article 6 of the Paris Agreement outlines general provisions, it lacks specific implementation guidelines. COP29 negotiations aim to operationalize Article 6 (especially 6.2 and 6.4), facilitating the regulation of global carbon credits.

### Energy transition

The GST at COP28 called for tripling renewable energy capacity and doubling energy efficiency globally by 2030.^1^ COP29 focused on implementing this energy transition goal, with hydrogen emerging as a potential cost-effective renewable energy source. China and Saudi Arabia, in particular, highlighted hydrogen’s role in industrial decarbonization, setting the stage for collaborative hydrogen initiatives (Figure 1B), while developed and developing countries failed to reach a consensus regarding the implementation of GST.

### Climate mitigation and adaption

Building on the UAE Framework for Global Climate Resilience, COP29 prioritized mitigation and adaptation. Key action areas include the following.

Digital technology: leveraging digital technologies for climate mitigation, adaptation, and early warning systems (Figure 1C). The COP29 Green Digital Action Declaration aims to accelerate the adoption of these technologies.
Methane and non-CO_2_ GHG reduction: a summit convened by China, the United States, and Azerbaijan focused on accelerating the mitigation of all GHGs in 2035 NDCs and net-zero targets (Figure 1D). The COP29 Declaration on Reducing Methane from Organic Waste complements the Global Methane Pledge (GMP), and a global nitrous oxide (N_2_O) assessment was, for the first time, launched to address climate mitigation and ozone depletion.
Climate-resilient agriculture: recognizing agriculture’s role as both a significant GHG source and a sector highly vulnerable to climate change, the COP29 Presidency launched the Baku Harmoniya Climate Initiative for Farmers to support the development of climate-resilient agrifood systems, balancing UN Sustainable Development Goals (SDGs), especially SDG2 and SDG13.
MAPs: Given cities' significant contributions to global CO_2_ emissions (over 67%),^2^ multisectoral action pathways (MAPs) emerged as a promising solution for sustainable urbanization (Figure 1E). The COP29 MAP Declaration for Resilient and Healthy Cities and the Declaration on Enhanced Action in Tourism were launched to support its implementation.

### Human development

Climate change poses a significant threat to human development, potentially displacing up to 1.2 billion people by 2050.^3^ Fragile populations, already facing humanitarian challenges, have limited capacity to adapt to climate change. Between 2014 and 2021, fragile states received an average of just $11 per person, compared to $162 per person for more stable contexts.^4^ To address these challenges, COP29 launched the COP Truce Appeal, calling for conflict prevention, support for vulnerable populations, and peacebuilding efforts. Additionally, the COP29 Presidency emphasized the importance of climate-positive education for children and youth (Figure 1F).

## Remaining challenges

While COP29 made significant progress in climate finance and action, several challenges persist.

Scientific assessment: the economic impacts of climate change are profound and complex. A quantitative assessment system is needed to understand and address these impacts, considering both direct and indirect effects on various sectors. These assessments will form the cornerstone for evidence-based policy formulation and the setting of targeted objectives. Furthermore, integrating climate change mitigation analysis is essential to inform a more feasible and accurate selection of mitigation strategies.
Policy consistency: intermittent governance and climate policies can weaken climate commitments and actions. For example, even though President Joe Biden rejoined the Paris Agreement and made ambitious commitments, the next administration of Donald J. Trump would very likely attempt to reverse much of the progress made. Ensuring consistent climate-positive policies regardless of political changes is crucial for long-term success.
Climate legislation: the legal regulatory framework for climate finance is underdeveloped compared to other economic sectors. Swift action is needed to establish robust climate legislation.
Practical actions: regardless of the very ambitious initiatives and launches by the COP29 Presidency, actual negotiations failed to achieve desirable results. For example, the COP Truce Appeal was signed by 132 parties, but almost no Western countries joined it, and the Russia-Ukraine war even escalated recently. We need prompt and practical actions.

## The journey to COP30

COP30 will be held in Belém, Brazil—home to the Amazon rainforest—in November 2025, a critical midpoint toward achieving the UN’s SDGs by 2030. As a precursor to COP30, the Legal Amazon Consortium has called for immediate actions to protect this vital ecosystem during COP29 (Figure 1G). This conference will be crucial for assessing new NDCs, promoting financial support for developing countries, accelerating the establishment of a global carbon market, and phasing out fossil fuels.

## Conclusion

COP29 marked a significant step forward in global climate action, while substantial challenges remain. The World Meteorological Organization (WMO) just reported that the global mean surface air temperature from January to September 2024 was 1.54°C above the pre-industrial average.^5^ Immediate actions must be taken to strengthen international cooperation and accelerate climate action in order to build a sustainable and resilient future for all.

## Acknowledgments

This study was supported by the National Natural Science Foundation of China (42261144687, 42203004), the 10.13039/100010097China Association for Science and Technology (CAST) United Nations Consultative Environmental Special Committee 2023 Project. We would like to thank Dr. Bridget Blake (Georgia State University, United States), Dr. Linda Anne Stevens (Asia-Pacific Network for Global Change Research), Dr. Xiaofeng Xu (China Meteorological Service Association), Prof. Yinyan Cai (Shenzhen Institute of Meteorological Innovation, China), Dr. Qianggong Zhang (Institute of Tibetan Plateau Research, Chinese Academy of Sciences), Dr. Xian Zhang (The Administrative Center for China’s Agenda 21), Dr. Wentao Wang (The Administrative Center for China’s Agenda 21), and Prof. Buda Su (School of Geographical Science, Nanjing University of Information Science & Technology, China) for their kind support in processing and discussing key issues at COP29.

## Declaration of interests

The authors declare no competing interests.
